# Effectiveness and safety of PD-1/PD-L1 inhibitors in advanced or recurrent endometrial cancer: a systematic review and meta-analysis

**DOI:** 10.3389/fphar.2023.1330877

**Published:** 2023-12-14

**Authors:** Songfei Han, Cuishan Guo, Zixuan Song, Ling Ouyang, Yizi Wang

**Affiliations:** Department of Obstetrics and Gynecology, Shengjing Hospital of China Medical University, Shenyang, China

**Keywords:** endometrial cancer, PD-1 inhibitors, PD-L1 inhibitors, immunity inhibitor, gynecological tumor

## Abstract

**Background:** Studies in recent years have shown that PD-1/PD-L1 inhibitors may have better effectiveness in patients with advanced or recurrent endometrial cancer. The effectiveness of PD-1/PD-L1 inhibitors is thought to be related to mismatch repair-deficient (dMMR) and mismatch repair-proficient (pMMR) classification in advanced or recurrent endometrial cancer. This study aims to evaluate the effectiveness of PD-1/PD-L1 inhibitors in patients classified as dMMR and pMMR.

**Methods:** Medical databases were searched to identify relevant publications up to 30 November 2022. The primary outcome was comparison of objective response rate (ORR) in patients with dMMR and pMMR following treatment with PD-1/PD-L1 inhibitors; secondary outcomes were single-group ORR in patients with dMMR and in patients with pMMR, respectively.

**Results:** Eleven studies were eligible for analysis and patients with advanced or recurrent endometrial cancer with molecular classification of dMMR had a higher total ORR than those with pMMR [odds ratio (OR), 7.70; 95% confidence interval (CI), 3.22–18.38; *p* < 0.01], with low evidence of between-study heterogeneity (I^2^ = 0%). The total ORR of patients with advanced or recurrent endometrial cancer with molecular type dMMR was 51.9% (95% CI, 33.6%–69.9%). The overall ORR of patients with advanced or recurrent endometrial cancer with molecular type pMMR was 16.1% (95% CI, 5.5%–30.3%).

**Conclusion:** In our including studies, the patients with advanced or recurrent endometrial cancer with molecular types of dMMR and pMMR, following treatment with PD-1/PD-L1 inhibitors, the total ORR of patients with dMMR was higher than that of patients with pMMR. Since the current number of studies is not very large, it is possible that more studies will be published in the future and more precise results will be discussed further.

## 1 Introduction

Endometrial cancer is the second most common malignant tumor of the female genital system worldwide (Siegel et al., 2023). It is estimated that there will be 66,200 new cases and 13,030 deaths due to endometrial cancer in 2023 in the United States (Siegel et al., 2023). Although most patients are diagnosed with endometrial cancer in its early stages, approximately 15% suffer advanced-stage disease (Brooks et al., 2019). The incidence of endometrial cancer is increasing due to the higher prevalence of obesity (Engerud et al., 2020). At present, treatment of endometrial cancer is primarily surgery, and the prognosis is generally good (Engerud et al., 2020). However, approximately 15%–20% of patients experience recurrence (Engerud et al., 2020). For patients with advanced or recurrent endometrial cancer, not only is the prognosis poor, but the treatment methods are very limited (Rousset-Rouviere et al., 2021). It is reported that patients with advanced or recurrent endometrial cancer have a 5-year survival rate of only 20% (Rousset-Rouviere et al., 2021) while the five-year survival rate for advanced breast cancer is as high as 29.1% (Mangone et al., 2022). Currently, the standard first-line treatment for advanced or recurrent endometrial cancer is chemotherapy with carboplatin and paclitaxel, but the response rate is only 10%–15% (Mathews et al., 2022). Of the other methods, only gestrol acetate is allowed for palliative care of advanced or recurrent endometrial cancer (Mathews et al., 2022). Recent studies have shown that immune checkpoint inhibitors (ICIs) have become an effective treatment strategy for advanced or recurrent endometrial cancer (Antill et al., 2021; Bellone et al., 2021; Hollebecque et al., 2021; Konstantinopoulos et al., 2022; Manning-Geist et al., 2022).

Programmed cell death protein-1 (PD-1) inhibitors and programmed cell death ligand-1 (PD-L1) inhibitors are types of immunologic drugs. PD-1/PD-L1 inhibitors bind PD-1 and PD-L1, respectively, to prevent or reverse exhausted T cells, thereby enhancing the anti-tumor immune mechanism (Acurcio et al., 2022). The Phase II KEYNOTE-158 study has shown that PD-1/PD-L1 inhibitors result in obvious improvement in many advanced cancers, such as advanced ovarian cancer, advanced lung cancer, and advanced kidney cancer (Marabelle et al., 2020). However, in the current studies, the effectiveness in advanced or recurrent endometrial cancer, the effects are maybe unclear.

It has been reported that microsatellite instability (MSI) in advanced or recurrent endometrial cancer is related to the efficacy of PD-1/PD-L1 inhibitors (O'Malley et al., 2022). Previous literature reported that 25%–31% of endometrial cancer patients had high levels of microsatellite instability (MSI-H) and mismatch repair-deficient (dMMR) (O'Malley et al., 2022).

In 2017, Ott et al. first published an evaluation of the effects of PD-1/PD-L1 inhibitors in patients with advanced or recurrent endometrial cancer typed as dMMR and mismatch repair-proficient (pMMR), and showed that the objective response rate (ORR) of patients with dMMR reached 100%, but the ORR of patients with pMMR was only 5.6% (Ott et al., 2017). However, the number of patients included in the study was small, with only 19 patients. Although the following studies highlighted the potential effectiveness of PD-1/PD-L1 inhibitors in patients with advanced or recurrent endometrial cancer, the results were less inconclusive. Therefore, the aim of this meta-analysis was to evaluate the effectiveness of PD-1/PD-L1 inhibitors in patients classified as dMMR and pMMR.

## 2 Methods

### 2.1 Literature Search and eligibility criteria

This meta-analysis was performed according to the Preferred Reporting Items for Systematic Reviews and Meta-Analyses (PRISMA) guidelines (Liberati et al., 2009), (Supplementary Table S1) and a literature search was performed with Embase, PubMed, Web of Science, and Cochrane databases up to 30 November 2022. Relevant studies were collected and duplicates removed for further screening (Identification). Based on the titles and abstracts, relevant studies were selected for full-text review (Screening). Based on the inclusion and exclusion criteria (Eligibility), we screened the studies for our meta-analysis (Included). If multiple studies reported the same outcomes based on the same patient population or overlapping information, we only included the most informative study. An additional search was performed among the references of the included studies to identify additional potentially eligible studies. After an initial comprehensive search and exclusion, 11 studies that met the inclusion criteria were identified (Konstantinopoulos et al., 2022; Bellone et al., 2021; Antill et al., 2021; O'Malley et al., 2022; Ott et al., 2017; Post et al., 2022; Wei et al., 2022; Makker et al., 2020; Pineda et al., 2020; Konstantinopoulos et al., 2019; Oaknin et al., 2022). The comprehensive search strings were “endometrial cancer,” “PD-1 inhibitors,” and “PD-L1 inhibitors.” Searches were performed without any restriction on publication year, but the language was limited to English. This meta-analysis was registered at PROSPERO (CRD4203248724).

Studies were included if they met the following inclusion criteria in accordance with PICOS (population, intervention, comparison, outcomes and study design) guidelines: 1: patients diagnosed with advanced or recurrent endometrial cancer; 2) patients who took PD-1/PD-L1 inhibitors as immunotherapy; 3) comparisons were made in patients with advanced or recurrent endometrial cancer with dMMR and pMMR; 4) comparisons were made of ORR in patients with advanced or recurrent endometrial cancer with molecular type dMMR and pMMR; and 5) studies were designed as prospective or retrospective cohort studies, case-control studies, or randomized controlled trials (RCTs).

The exclusion criteria were as follows: (1) the number of study cases was less than five patients; (2) comments or reviews; (3) preclinical experiments; and (4) case reports.

### 2.2 Data collection and outcome measures

Data were extracted from each paper using a standardized table: 1) authors; 2) study design; 3) total number of patients; 4) research setting; 5) number of enrolled cases; 6) age of the patient; 7) year of publication; 8) effective rate of the drug in clinical application; 9) history of previous medical records and medications; 10) the ORR of pMMR and dMMR before and after PD-1/PD-L1 inhibitors; 11) number and type of adverse event events; 12) follow-up period (Table 1). The quality of the studies was independently assessed by two reviewers using the Newcastle-Ottawa Scale (NOS) (Table 2) (Stang, 2010). The certainty of the evidence was assessed according to GRADE guidelines (Foroutan et al., 2020). According to the predefined criteria, two investigators independently screened all the relevant studies and reviewed the full texts of the included studies. If there was a disagreement, it was discussed and solved by consensus with a third reviewer.

**TABLE 1:** Characteristics of the individual studies and enrolled population.

Study	Country	Age (years)	Drug	Study Period	Follow-up (median-months)	No. of Prior chemotherapy (*n*)	Setting	Total (*n*)	No. of dMMR (*n*)	No. of pMMR (*n*)
1. Ott et al. (2017)	US	Median 67 (range 34–87)	PEM	NA	76.2	12	Multi	24	1	18
2. Konstantinopoulos et al. (2019)	US	NA	AVE	2016–2018	18.6	25	Multi	31	15	16
3. Makker et al. (2020)	US	Mean 65.3	LEN+PEM	2015–2018	18.7	108	Multi	124	11	94
4. Pineda et al. (2020)	US	Median 67 (range 43–86)	PEM+CAR+PAC	NA	NA	46	Multi	46	NA	NA
5. Bellone et al. (2021)	US	NA	PEM	2016–2020	25.8	25	Multi	24	24	0
6. Antill et al. (2021)	US	Median 67 (range 36–81)	DUR	2017–2018	8.3 (dMMR) 14.8 (pMMR)	71	Multi	71	35	36
7. O’Malley et al. (2022)	US	Median 64 (range 42–86)	PEM	2016–2020	NA	90	Multi	90	90	0
8. Oaknin et al. (2022)	US	Median 64.5 (range 58.5–69.5) (dMMR) Median 64.5 (range 30–86) (pMMR)	DOS	2017–2020	16.3 (dMMR) 11.5 (pMMR)	264	Multi	256	108	156
9. Konstantinopoulos et al. (2022)	US	Mean 67.9	AVE+TAL	2019–2020	12.9	35	Multi	35	0	35
10. Post et al. (2022)	Netherlands	Median 69 (range 64.3–73.0)	DUR+OLA	2019–2020	17.6	42	Multi	50	10	NA
11. Wei et al. (2022)	China	Median 56 (range 37–70)	SIN+ANL	2019–2020	15.4	11	Single	23	9	14

AVE, avelumab; TAL, talazoparib; DUR, durvalumab; OLA, olaparib; SIN, sintilimab; ANL, anlotinib; PEM, pembrolizumab; LEN, lenvatinib; CAR, carboplatin; PAC, paclitaxel; DOS, dostarlimab.

**TABLE 2:** Quality assessment of the included studies according to the Newcastle–Ottawa scale.

Study	Selection				Outcome assessment			Comparability Score
	Representativeness of cohort	Selection of non-exposedcohort	Ascertainment of exposure	Absence of outcome at baseline	Assessment of Length of outcome	follow-up	Adequacy of follow-up	
1. Ott et al. (2017)	*		*	*	*	*	*	6
2. Konstantinopoulos et al. (2019)	*		*	*	*	*	*	6
3. Makker et al. (2020)	*		*	*	*	*	*	6
4. Pineda et al. (2020)	*		*	*	*	*		5
5. Bellone et al. (2021)	*		*	*	*	*	*	6
6. Antill et al. (2021)	*		*	*	*	*	*	6
7. O’Malley et al. (2022)	*	*	*	*	*	*	*	7
8. Oaknin et al. (2022)	*		*	*	*	*		5
9. Konstantinopoulos et al. (2022)	*		*	*	*	*	*	6
10. Post et al. (2022)	*		*	*	*	*		5
11. Wei et al. (2022)	*		*	*	*	*	*	6

### 2.3 Statistical analysis

We extracted ORR and 95% confidence intervals (CIs) from all included studies. We also calculate the odds ratio (OR) to evaluate the patients’ severe toxicity profile (G3-G4 toxicity) of the PD-1/PD-L1 inhibitors. The meta-analysis was performed with R 4.3.0 and Review Manager 5.3, and the pooled ORR was calculated using random-effects models to reduce the heterogeneity between studies (DerSimonian and Laird, 2015). Heterogeneity between studies was evaluated with the χ2 test and I^2^ statistic, and I^2^ values of less than 25%, 25%–75%, and greater than 75% were considered low, moderate, and high heterogeneity, respectively (Higgins et al., 2003). The robustness of the main findings were assessed using sensitivity analyses (Copas and Shi, 2000). We also performed subgroup analyses to identify sources of heterogeneity. Funnel plots with Begg’s and Egger’s regressions were used to examine the effect of publication bias (Begg and Mazumdar, 1994; Vandenbroucke et al., 1998). A *p*-value less than 0.05 was considered significant.

## 3 Results

### 3.1 Study selection

**FIGURE 1:**
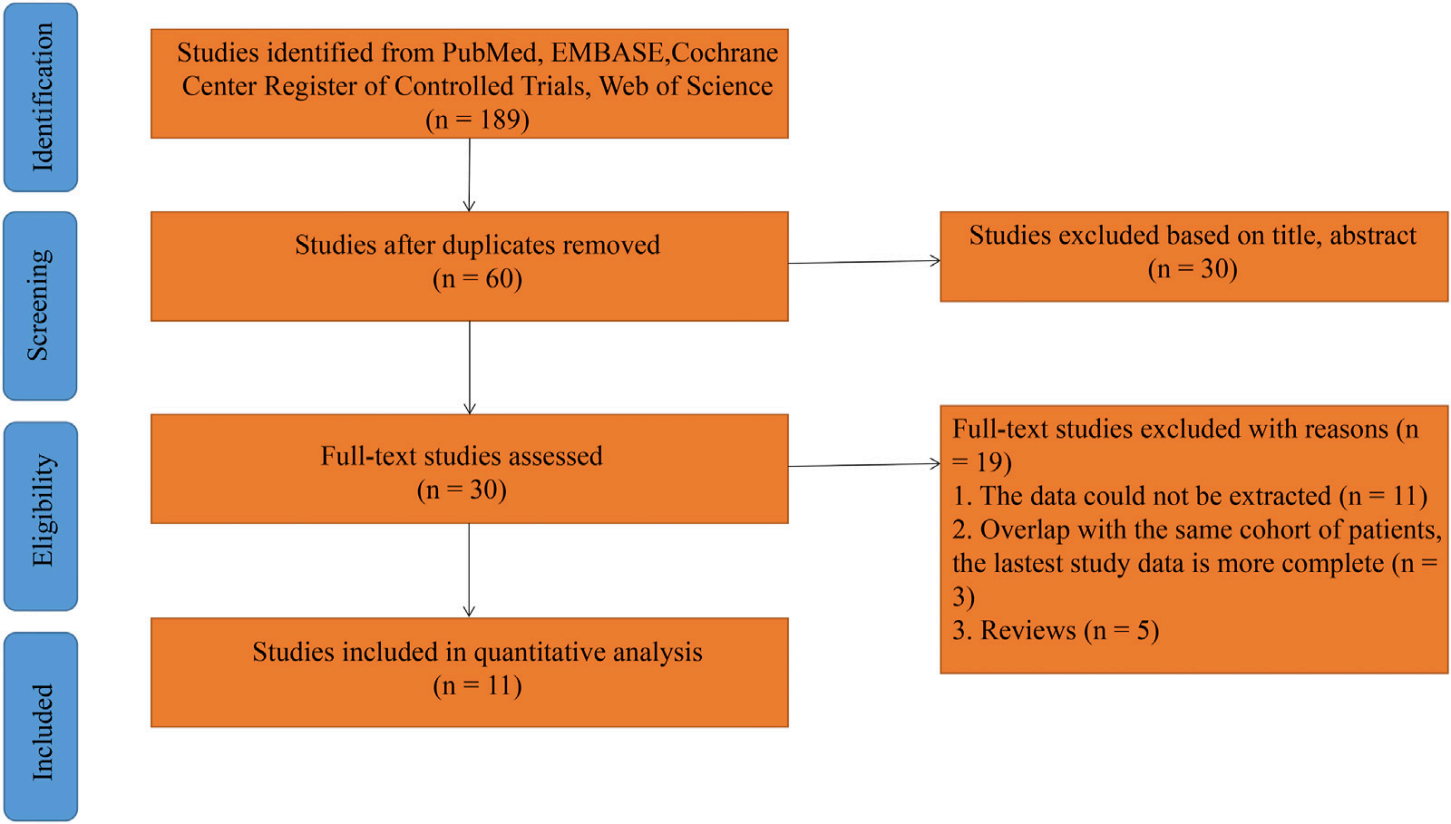
Flow chart of this meta-analysis.

The flow chart of this meta-analysis is presented in Figure 1. In total, 189 studies were identified in PubMed, EMBASE, Cochrane Central Register of Controlled Trials and Web of Science databases. According to the abstracts or titles of the articles during preliminary screening, 29 full-text papers and one meeting abstract were further scrutinized. Eleven publications were excluded because they did not provide data (Marcus et al., 2019; Rubinstein et al., 2019; Walsh et al., 2019; Hollebecque et al., 2021; Oaknin et al., 2021; Kristeleit et al., 2022; Liao et al., 2022; Mimura et al., 2022; Babar et al., 2023; Chow et al., 2023; Dioun et al., 2023); three publications were excluded because they overlapped with the same cohort of patients, and the latest study data was more complete (Oaknin et al., 2020a; Oaknin et al., 2020b; Marabelle et al., 2020); and five publications were excluded because they were reviews (Sobecki-Rausch and Barroilhet, 2019; Kasherman et al., 2021; Costa and Vale, 2022; Turinetto et al., 2022; Walker et al., 2023). Finally, a total of 11 articles were included in the meta-analysis. Nine studies in our included study were from the United States (Konstantinopoulos et al., 2022; Bellone et al., 2021; Antill et al., 2021; O’Malley et al., 2022; Ott et al., 2017; Makker et al., 2020; Pineda et al., 2020; Konstantinopoulos et al., 2019; Oaknin et al., 2022). One study was from the Netherlands (Post et al., 2022) and one study was from China (Wei et al., 2022). Of the 11 studies, one study included patients with pMMR (Konstantinopoulos et al., 2022), two studies include patients with dMMR (Bellone et al., 2021; O’Malley et al., 2022), one study focused on the number and types of adverse reactions (Pineda et al., 2020), and the remaining seven studies included patients with both pMMR and dMMR (Ott et al., 2017; Konstantinopoulos et al., 2019; Makker et al., 2020; Antill et al., 2021; Oaknin et al., 2022; Post et al., 2022; Wei et al., 2022). A total of 369 patients with pMMR and 292 patients with dMMR were included in the meta-analysis. Six of the studies were single-agent studies, including pembrolizumab (Bellone et al., 2021; O’Malley et al., 2022; Ott et al., 2017), avelumab (Konstantinopoulos et al., 2019), durvalumab (Antill et al., 2021), and dostarlimab (Oaknin et al., 2022); five studies were combined treatments, including talazoparib and avelumab (Konstantinopoulos et al., 2022), durvalumab and olaparib (Post et al., 2022), sintilimab and anlotinib (Wei et al., 2022), lenvatinib and pembrolizumab (Makker et al., 2020), and pembrolizumab, carboplatin, and paclitaxel (Pineda et al., 2020).

Of the 11 articles, 10 articles were multiple center (Konstantinopoulos et al., 2022; Bellone et al., 2021; Antill et al., 2021; O’Malley et al., 2022; Ott et al., 2017; Post et al., 2022; Makker et al., 2020; Pineda et al., 2020; Konstantinopoulos et al., 2019; Oaknin et al., 2022) and one article was single center (Wei et al., 2022). One of the papers was not a full-text document, but a meeting abstract (Pineda et al., 2020).

### 3.2 Effectiveness of PD-1 and PD-L1 immune checkpoint inhibitors on dMMR and pMMR

**FIGURE 2:**
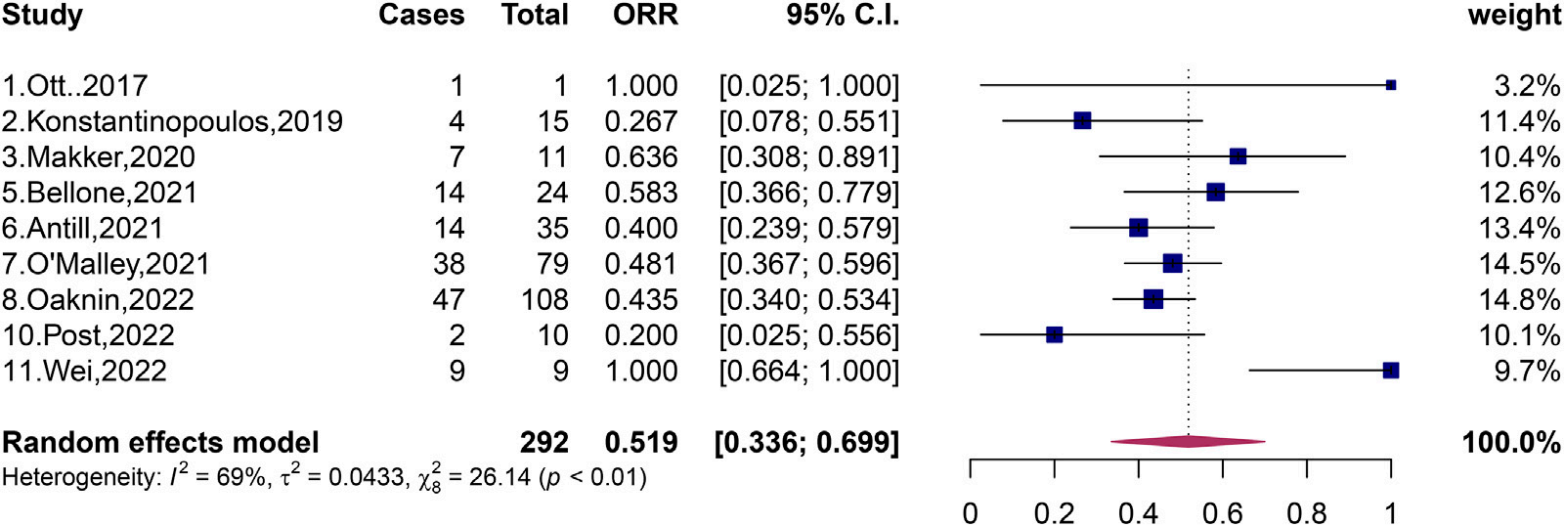
Forest plots of the included studies evaluating efficacy of PD-1/PD-L1 inhibitors to patients with dMMR.

**FIGURE 3:**
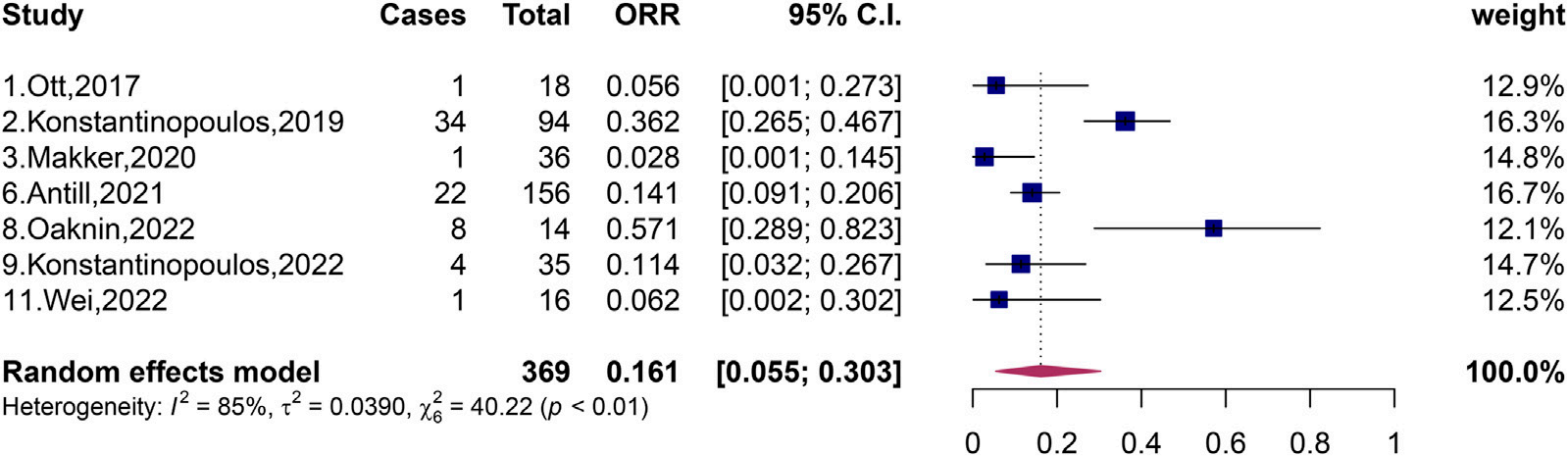
Forest plots of the included studies evaluating efficacy of PD-1/PD-L1 inhibitors to patients with pMMR.

A total of 369 patients with pMMR and 292 patients with dMMR were included in the 11 studies, and the total ORR of patients with dMMR was 51.9% (95% CI, 33.6%–66.9%; I^2^ = 69%, *p* < 0.01) (Figure 2). The total ORR of patients with pMMR was 16.1% (95% CI, 5.5%–30.3%; I^2^ = 85%, *p* < 0.01) (Figure 3). These results suggested that dMMR may be more sensitive than pMMR.

### 3.3 Comparison of PD-1/PD-L1 inhibitors in patients with advanced or recurrent endometrial cancer typed as dMMR and pMMR

**FIGURE 4:**
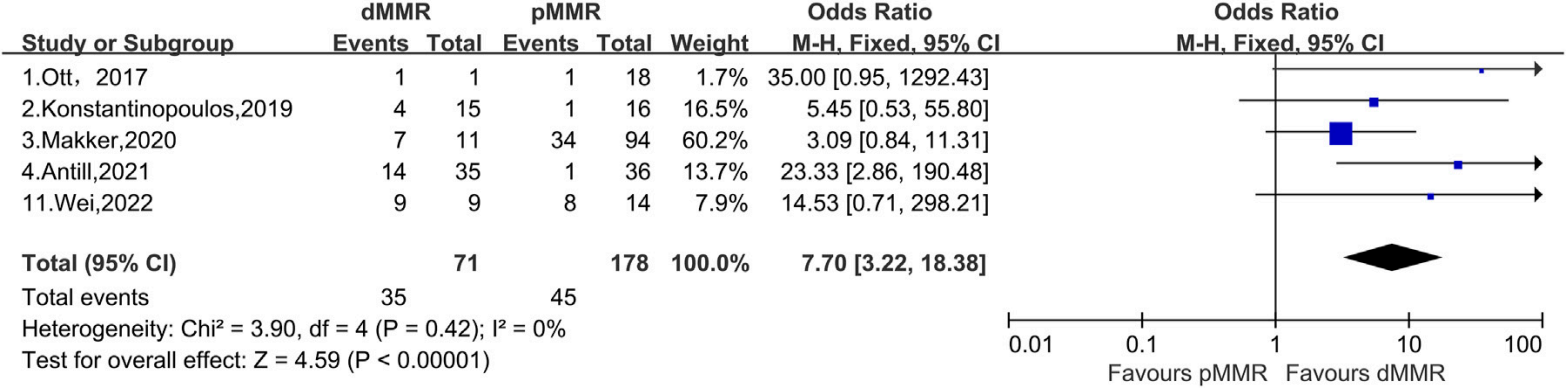
Forest plots of the included studies evaluating efficacy of the comparison of PD-1/PD-L1 inhibitors to patients with dMMR and pMMR.

We compared patients with pMMR and dMMR before and after treatment with PD-1/PD-L1 inhibitors, which was a total of 71 patients with dMMR and 178 patients with pMMR with advanced or recurrent endometrial cancer. In our further analysis of ORR in patients with dMMR and pMMR, we found that the OR for the effect of PD-L1 inhibitors was 7.70 (95% CI, 3.22%–18.32%) (Figure 4). Furthermore, we found that the 95% CI published by Ott et al. reached 0.95%–1,292.43% (Ott et al., 2017); by contrast, Makker et al. showed a 95% CI of only 0.84%–11.31% (Makker et al., 2020); these Cis clearly vary greatly. The combined heterogeneity of these five studies was I^2^ = 0% (*p* = 0.42). The results after our analysis showed that treatment with PD-1/PD-L1 inhibitors was more effective in patients with dMMR than patients with pMMR.

### 3.4 Safety of immunosuppressants in the treatment of advanced or recurrent endometrial cancer

We included 11 studies in which a total of 774 patients received treatment with PD-1/PD-L1 immune checkpoint inhibitors (ICIs). All included studies of adverse events (Table 3). Eleven studies had a total of 68 grade 3/4 adverse effects. We statistics that the most common and the highest incidence was hypertension (5.65%), anemia (4.26%), fatigue (2.58%), and acral redness and swelling and severe skin reactions (2.58%). Oaknin et al. (2022) showed that patients with advanced or recurrent endometrial cancer classified as dMMR had a probability of grade 3/4 adverse reactions of only 13.2% (17/129); by contrast, patients with advanced or recurrent endometrial cancer classified as pMMR had a probability of grade 3/4 adverse reactions of 19.3% (31/161). In our results, patients classified as dMMR had a high probability of anemia (3.9%) and lipase increase (2.3%), and did not have symptoms of hyperglycemia and fatigue while patients with recurrent advanced endometrial cancer classified as pMMR developed fatigue (2.5%) and hyperglycemia (1.9%), with a probability of only 1.9% of anemia and a 1.9% probability of increased lipase (Oaknin et al., 2022).

**TABLE 3:** Treatment- related adverse events of grade 3/4 in all patients.

	Preferred term or basket	Patient (*n* = 774)
		Grade 3/4, *n*(%)
Gastrointestinal system
	Diarrhea	15(1.94%)
	Lipase increased	11(1.42%)
	Nausea	6(0.78%)
	Amylase increased	6(0.78%)
	Colitis	5(0.65%)
	Hepatotoxicity and hepatitis	5(0.65%)
	Alanine aminotransferase increased	4(0.52%)
	Aspartate transaminase increased	3(0.39%)
	Transaminases increased	2(0.26%)
	GI perforation	2(0.26%)
	Gastrointestinal	2(0.26%)
	Constipation	2(0.26%)
	Colitis ischemic	2(0.26%)
	Abdominal pain and upper abdominal pain	2(0.26%)
	Vomit	1(0.13%)
	Small intestinal obstruction	1(0.13%)
	Rectal ulcer	1(0.13%)
	Peritonitis	1(0.13%)
	Cholecystitis acute	1(0.13%)
	Back pain	1(0.13%)
	Pancreatitis	1(0.13%
Endocrine/metabolism
	Hyponatremia	6(0.78%)
	Adrenal insufficiency	4(0.52%)
	Hyperglycemia	4(0.52%)
	Hypothyroidism	2(0.26%)
	Hypomagnesemia	2(0.26%)
	Hypokalemia	2(0.26%)
	Dehydration	2(0.26%)
	Hypophysitis	1(0.13%)
	Hypocalcemia	1(0.13%)
	Hypertriglyceridemia	1(0.13%)
	Hyperkalemia	1(0.13%)
	Type 1 diabetes mellitus	1(0.13%)
General
	Hypertension	42(5.65%)
	Fatigue	20(2.58%)
	Asthenia	9(1.16%)
	Syncope	4(0.52%)
	Hemorrhage	4(0.52%)
	Anaphylaxis	3(0.39%)
	Fever	3(0.39%)
	Weight decreased	2(0.26%)
	Abscess	2(0.26%)
	Oral pain	1(0.13%)
	Hypotension	1(0.13%)
	Dysarthri	1(0.13%)
	Anorexia	1(0.13%)
Musculoskeletal system
	Pain and arthralgia	2(0.26%)
	Myocarditis	2(0.26%)
	Creatinine increased	2(0.26%)
	Myalgia	1(0.13%)
Respiratory system
	Pulmonary embolism	6(0.78%)
	Pneumothorax	1(0.13%)
	Dyspnea	1(0.13%)
Nervous system
	Confusional state and deliriuml	4(0.52%)
	Neurological	2(0.26%)
Hematologic system
	Anemia	33(4.26%)
	Platelet count decreased	11(1.42%)
	Neutrophil count decreased	7(0.90%)
	Escherichia sepsis	1(0.13%)
Cardiovascular system
	QT prolongation and cardiac dysfunction	3(0.39%)
	Sinus bradycardia	1(0.13%)
	Arterial TE events	1(0.13%)
Renal system
	Proteinuria	5(0.65%)
	Renal events and nephritis	2(0.26%)
Laboratory abnormalities		20(2.58%)
PPE and severe skin reactions		16(2.08%)
Immune- related event		7(0.90%)
Others		6(0.78%)

## 4 Discussion

Advanced or recurrent endometrial cancer has a poor prognosis and a high recurrence rate of approximately 40%–70% (Tronconi et al., 2022). Treatment of advanced or recurrent endometrial cancer is unsatisfactory and options are limited (Brooks et al., 2019). At present, the conservative treatment of advanced or recurrent endometrial cancer is hormone therapy and paclitaxel plus carboplatin chemotherapy, and the response rate is low (Gadducci and Cosio, 2021). Therefore, identifying new treatments to address advanced or recurrent endometrial cancer has become important. In recent years, immunotherapy has become a hot spot in the treatment of advanced or recurrent endometrial cancer.

Programmed cell death protein-1 (PD-1) is a protein found on the surface of T cells and is expressed in 90% of endometrial cancers (Antill et al., 2021). Programmed cell death ligand-1 (PD-L1) binds PD-1, PD-1 is phosphorylated by protein tyrosine kinase (Lck) and recruits the tyrosine kinase Shp2 (Src homologous phosphatase 2), when accumulated to a certain extent, it dephosphorylates the T-cell receptor (TCR) and CD28, and consequently, T-cell function and signalling are inhibited, however, when PD-1/PD-L1 inhibitors intervene, PD-1 in the cell membrane cannot be phosphorylated, resulting in the cells not being able to recruit to Shp2, which in turn leads to the TCR and CD28 not being phosphorylated, and the activated immune signals can be transmitted smoothly, and the T cells proliferate and differentiate. Eventually T cells are activated (Hui et al., 2017; Kamphorst et al., 2017; Liu et al., 2019; Xia et al., 2019). PD-1/PD-L1 inhibitors do not kill cancer cells themselves, but block the binding of PD-1 and PD-L1, thereby blocking the transmission of inhibitory signals mediated by these molecules, resulting in activation of T cells, enhancing the patient immune defense mechanism, and playing an anti-tumor role (Liao et al., 2022). Patients with advanced or recurrent endometrial cancer classified as dMMR may be more sensitive to PD-1/PD-L1 inhibitors because these patients have higher expression of PD-1 and PD-L1 in the TME than patients with pMMR (Gatalica et al., 2014). Further experiments have revealed that following treatment with PD-1/PD-L1 inhibitors, the ORR is 46% for patients with advanced or recurrent endometrial cancer with molecular type dMMR, and 13% for patients with advanced or recurrent endometrial cancer with molecular type pMMR (Rizzo, 2022). However, further studies may be needed to explore whether PD-1/PD-L1 inhibitors are more effective in patients with dMMR compared to patients with pMMR.

Four studies reported the effectiveness of single-agent PD-1/PD-L1 inhibitors in patients in advanced or recurrent endometrial cancer with pMMR (Ott et al., 2017; Konstantinopoulos et al., 2019; Antill et al., 2021; Oaknin et al., 2022). Results from these studies suggest that PD-1/PD-L1 inhibitors may be less effective in patients with pMMR. In 2017, Ott et al. published the first study on the efficacy of single-agent pembrolizumab in the treatment of advanced endometrial cancer, and concluded that the total ORR of patients was 13.0% (3/23, 95% CI, 2.8%–33.6%), all of which were cases of partial response (Ott et al., 2017). In 2019, Konstantinopoulos et al. studied the efficacy of single-agent avelumab in the treatment of recurrent endometrial cancer classified as pMMR and showed that the ORR of patients with recurrent endometrial cancer with molecular type pMMR was 6.25% (1/16, 95% CI, 0.16%–30.2%); all were partial responses (Konstantinopoulos et al., 2019). In 2021, Antill et al. studied the efficacy of durvalumab in patients with advanced endometrial cancer. Their experimental results showed that patients with advanced endometrial cancer classified as pMMR had an ORR of only 3% (1/35, 95% CI, 1%–15%) (Antill et al., 2021). In 2022, Oaknin et al. evaluated the effects of single-agent dostarlimab in patients with advanced or recurrent endometrial cancer, including a large number of experimental subjects and 156 pMMR patients. The ORR of patients classified as pMMR was only 14.1% (22/156, 95% CI, 0.1%–20.6%), but rare cases of complete response occurred in patients with pMMR. The complete response rate (CR) was 1.9% and the partial response rate (PR) was 12.2% (Oaknin et al., 2022). All three experimental studies included the efficacy of monotherapy for advanced or recurrent endometrial cancer classified as pMMR, although the results were less unsatisfactory. The ORR was low; the highest was 14.1% (Oaknin et al., 2022) and the lowest was 3% (Antill et al., 2021).

In the following study, Makker, Wei and Konstantinopoulos et al. investigated the efficacy of combination of dual-agent in patients with pMMR (Makker et al., 2020; Konstantinopoulos et al., 2022; Wei et al., 2022), and the experimental data showed that the ORR was relatively higher than that of single-agent (Ott et al., 2017; Konstantinopoulos et al., 2019; Antill et al., 2021; Oaknin et al., 2022). In 2020, Makker et al. published an evaluation of the efficacy of the combination of dual-agent lenvatinib and pembrolizumab in advanced endometrial cancer classified as pMMR and showed that the ORR of patients classified as pMMR was 37.2% (35/94, 95% CI, 27.5%–47.8%) (Makker et al., 2020). Later, in 2022, Wei et al. published an evaluation report on the efficacy of the combination of sintilimab and anlotinib in the treatment of advanced or recurrent endometrial cancer classified as pMMR, and the ORR of pMMR patients was as high as 57.1% (8/14, 95% CI, 28.9%–82.3%). The ORR in this study was much higher than those of other pMMR patients in the literature we included, and patients with complete response of a rare type of pMMR occurred in small sample sizes, with CR of 7.1% and PR of 50% (Wei et al., 2022). There may be two reasons why the ORR in the Wei et al. study reached such a high level: first, the article included Asians, which was different from the population of the subjects included in other papers we included; second, anlotinib is proposed to improve the sensitivity of PD-1/PD-L1 inhibitors in patients with advanced or recurrent endometrial cancer typed as pMMR (Wei et al., 2022). In the same year, Konstantinopoulos et al. published a two-drug talazoparib and avelumab combination treatment of patients with recurrent endometrial cancer typed as pMMR, revealing an ORR of only 11.4% (4/35, 95% CI, 3.2%–26.7%); all were partial remission cases (Konstantinopoulos et al., 2022). In these three publications, patients with pMMR had a maximum ORR of 57.1% after PD-1/PD-L1 inhibitors (Wei et al., 2022); the minimum was 11.4% (Konstantinopoulos et al., 2022). After pooling patients with pMMR, we generated a forest plot showing that the total ORR of pMMR patients was 16.1% (95% CI, 5.5%–30.3%, *p* < 0.01), with large heterogeneity, and perhaps heterogeneity from the study published by Wei et al. (2022).

Recent studies have shown that PD-1/PD-L1 inhibitors may have a good effect on patients with advanced or recurrent endometrial cancer classified as dMMR (Bellone et al., 2021; Antill et al., 2021; O’Malley et al., 2022; Konstantinopoulos et al., 2019; Oaknin et al., 2022). We have included several studies on the efficacy of dMMR patients, and the results of these studies indicate that dMMR patients may be more sensitive to PD-1/PD-L1 inhibitors (Bellone et al., 2021; Antill et al., 2021; O’Malley et al., 2022; Konstantinopoulos et al., 2019; Oaknin et al., 2022). In 2019, Konstantinopoulos et al. published an evaluation of the effect of single-agent avelumab in the treatment of recurrent endometrial cancer classified as dMMR, which revealed an ORR of patients with recurrent endometrial cancer classified as dMMR as high as 26.7% (4/15, 95% CI, 7.8%–55.1%) and CR of 6.67% and PR of 20% (Konstantinopoulos et al., 2019). In 2021, Antill et al. published a study on the therapeutic effect of single-agent durvalumab in patients with advanced endometrial cancer, revealing patients with advanced endometrial cancer classified as dMMR had an ORR of up to 47% (17/36, 95% CI, 32%–63%), CR of 16.7%, and PR of 30.6% (Antill et al., 2021). The study published by Antill et al. showed that PD-1/PD-L1 inhibitors were very effective in patients with advanced endometrial cancer with dMMR, and nearly half of patients with dMMR had good results. In the same year, Bellone et al. also published an experimental study on single-agent pembrolizumab in the treatment of recurrent endometrial cancer classified as dMMR, revealing an ORR of patients with dMMR of 58% (14/24, 95% CI, 36.6%–77.9%) (Bellone et al., 2021). More than half of the patients had promising treatment results. In 2022, O'Malley et al. published an evaluation of the efficacy of single-agent pembrolizumab in patients with advanced endometrial cancer with molecular classification of dMMR. This study showed that the ORR of patients with dMMR reached 48% (38/79, 95% CI, 37%–60%) (O'Malley et al., 2022). In the same year, Oaknin et al. published a single-agent dostarlimab treatment evaluation of patients with advanced or recurrent endometrial cancer classified as dMMR, which showed that the ORR of patients with dMMR reached 43.5% (47/108, 95% CI, 34.0%–53.4%), CR was 10.2%, and PR was 33.3% (Oaknin et al., 2022). These five studies indicate a relatively high ORR, ranging from 26.7% (Konstantinopoulos et al., 2019) to 58% (Bellone et al., 2021). In these five stuides (Bellone et al., 2021; Antill et al., 2021; O’Malley et al., 2022; Konstantinopoulos et al., 2019; Oaknin et al., 2022), it seems that patients with advanced or recurrent endometrial cancer classified as dMMR may have been shown to be sensitive to PD-1/PD-L1 inhibitors and treatment has been more effective in this patients in monotherapy studies compared with patients with advanced or recurrent endometrial cancer classified as pMMR. In 2020, Makker et al. published an evaluation of the efficacy of the combination of dual-agent lenvatinib and pembrolizumab in the treatment of advanced endometrial cancer with molecular type dMMR, revealing an ORR of patients with dMMR as high as 63.6% (7/11, 95% CI, 30.8%–89.1%) (Makker et al., 2020). In 2022, Wei et al. evaluated the efficacy of the combination of dual-drug sintilimab and anlotinib in the treatment of advanced or recurrent endometrial cancer, revealing an ORR of patients with recurrent advanced endometrial cancer classified as dMMR as high as 100% (9/9, 95% CI, 64%–100%), CR of 22.2%, and PR of 77.8% (Wei et al., 2022). The experimental results of both studies (Makker et al., 2020; Wei et al., 2022) show that the ORR of patients with advanced or recurrent endometrial cancer classified as dMMR may be high in the case of two-agent combination therapy, indicating that patients classified as dMMR are sensitive to PD-1/PD-L1 inhibitors, resulting in significant efficacy, probably.

We then generated a forest plot of the efficacy of patients with dMMR after PD-1/PD-L1 inhibitor treatment, showing that the total ORR of patients with dMMR was 51.9% (95% CI, 33.6%–69.9%, *p* < 0.01), which was much higher than the total ORR of patients with pMMR. We further compared PD-1/PD-L1 inhibitors with dMMR or pMMR in patients with endometrial cancer and found an improved ORR (OR, 7.70; 95% CI, 3.22–18.38; *p* < 0.01) for endometrial cancer patients with dMMR receiving PD-1/PD-L1 inhibitors compared those with pMMR.

To further support our conjecture, we made a comparison of dMMR and pMMR. The total OR of patients with dMMR and pMMR was 7.70 (95% CI, 3.22–18.38; *p* < 0.01), indicating that patients with dMMR may be more sensitive to PD-1/PD-L1 inhibitors, resulting in much higher efficacy than patients with pMMR. The reason patients with advanced or recurrent endometrial cancer classified as dMMR are more sensitive to PD-1/PD-L1 inhibitors is probably because these patients have higher expression of PD-L and PD-L1 in the TME than patients with pMMR (Gatalica et al., 2014). With high expression of PD-1/PD-L1 in the TME, administration of PD-1/PD-L1 inhibitors results in a greater binding of the inhibitors and receptors, resulting in greater efficacy (Gatalica et al., 2014). Thus, the results after our analysis show that treatment of patients with advanced or recurrent endometrial cancer classified as dMMR with PD-1/PD-L1 inhibitors is more effective than patients with pMMR. However, one study reported that patients with dMMR are more likely to develop primary resistance with the use of PD-1/PD-L1 inhibitors, but the mechanism of resistance and its probability of occurrence have not been elucidated (Nebot-Bral et al., 2019).

Safety is an important aspect of all innovative studies, and all the studies included in this meta-analysis included adverse effects of treatment and their probabilities (Konstantinopoulos et al., 2022; Bellone et al., 2021; Antill et al., 2021; O’Malley et al., 2022; Ott et al., 2017; Post et al., 2022; Wei et al., 2022; Makker et al., 2020; Pineda et al., 2020; Konstantinopoulos et al., 2019; Oaknin et al., 2022). For patients who are obese and have hypertensive diseases, adverse events are more likely to occur (Wei et al., 2022). The single-agent study with the highest variety of grade 3/4 adverse reactions was the study on durvalumab published by Antill et al. (Antill et al., 2021); only one grade 3/4 adverse reaction, viral hepatitis, was reporter (Antill et al., 2021). The single-agent study with the largest variety of grade 3/4 adverse reactions was the study on dostarlimab published by Oaknin et al. (Oaknin et al., 2022), with 14 grade 3/4 adverse reactions, including anemia, thrombocytopenia, and vomiting. In this study (Oaknin et al., 2022), the probability of grade 3/4 adverse reactions in advanced or recurrent endometrial cancer classified as dMMR was only 13.2% (17/129) while the probability of grade 3/4 adverse reactions in patients with advanced or recurrent endometrial cancer classified as pMMR was as high as 19.3% (31/161). After our analysis, the grade 3/4 adverse events with a high probability of dMMR were anemia (3.9%) and increased lipase (2.3%), and no hyperglycemic and fatigue events occurred. However, patients with advanced or recurrent endometrial cancer classified as pMMR had grade 3/4 adverse effects, which were mainly fatigue (2.5%) and hyperglycemia (1.9%), anemia (1.9%), and lipase increase (1.9%). The combination study with the fewest grade 3/4 adverse effects was the study published by Konstantinopoulos et al. on talazoparib and avelumab (Konstantinopoulos et al., 2022) with four grade 3/4 adverse reactions, namely, anemia, thrombocytopenia, fatigue, and neutropenia. The combination drug study with the most types of grade 3/4 adverse reactions was the combination therapy study of lenvatinib and pembrolizumab published by Makker et al. (2020), which described high blood pressure, fatigue, and diarrhea. In the 11 studies included in this meta-analysis, different grade 3/4 adverse events were described. The final effective experimental sample was 774, and a total of 68 grade 3/4 adverse events occurred, including hypertension, anemia, vomiting, weight loss, fatigue, nausea, vomiting, and diarrhea, with a high probability of occurrence, namely, hypertension (5.65%), anemia (4.26%), fatigue (2.58%), diarrhea (1.94%), and thrombocytopenia (1.42%). However, these adverse reactions can be slowly alleviated by reducing the dose of the drug or discontinuing the medication (Oaknin et al., 2022). Timely detection of toxicological effects and adverse reactions caused by drugs, and timely reduction or even interruption of the dose of drugs, may be more effective in the treatment of cancer. For the moment, there are slight differences in adverse effects of different inhibitor drugs, but in general, the efficacy is significant and toxicity is controllable compared to other drugs.

### 4.1 Heterogeneity analysis

Of the 11 included studies, one was relatively heterogeneous compared with the others (Wei et al., 2022). It was a study of the combination of sintilimab and anlotinib. Firstly, we believe that the number of cases of study patients is much smaller than the other studies, which is a large part of the reason. The study included 23 cases, and the results of the single-center study may lead to a certain deviation. The patients were from China, and the research subjects of the other studies were from the Netherlands and the United States, which may result in ethnic differences and inconsistencies in the level of various biochemical indicators of the human body. Its results show both pharmacological and toxicological effects had clear effects, with high rates of response (ORR 73.9%) and high incidence of adverse events (all participants experienced adverse events of different levels). Another study with 95% CI ranging from 0.95 to 1,292.43 had a large difference in the intervals (Ott et al., 2017), but after combining this study with the others, the pooled heterogeneity was insignificant (I^2^ = 0). We suspect that only one dMMR patient was included in this study with a 100% efficiency rate, whereas the number of pMMR patients included was 18, but the efficiency rate was only 5.6%. The number of dMMR patients included was much smaller than that of pMMR patients, leading to a larger difference in confidence intervals. The results were consistent with other studies; both patients with dMMR showed higher efficiency with PD-1/PD-L1 inhibitors, thus the heterogeneity after combination was insignificant. Hence, we did not perform sensitivity analysis. In the future, multicenter studies, especially in Asian populations, may provide a more comprehensive assessment of the efficacy of PD-1/PD-L1 inhibitors for recurrent endometrial cancer.

### 4.2 Limitation

This is the first meta-analysis to comprehensively analyze the available data on PD-1/PD-L1 inhibitors for the treatment of endometrial cancer patients typed as dMMR and pMMR in clinical practice. The results suggest that patients with dMMR are more sensitive to PD-1/PD-L1 inhibitors. However, several limitations of this meta-analysis should be considered. Firstly, we are limited by the characteristics of some tumors, such as tumor pathological type and FIGO stage, because not every study analyzed the effect of PD-1/PD-L1 inhibitors in patients with different stages and pathological types. Thus, our meta-analysis cannot elaborate these tumor characteristics, resulting in a certain bias. Secondly, the small sample size and single-arm design hinder the generality of our findings, which may cause certain selection or information biases, and making it difficult to draw objective clinical efficacy and concrete conclusions. Finally, because the immunosuppressant PD-1/PD-L1 drugs used by patients are different in different research projects, this can lead to some bias, and it is difficult to calculate the effect of each drug separately.

## 5 Conclusion

Our results suggest that the therapeutic efficacy of PD-1/PD-L1 inhibitors is associated with the molecular typing of advanced or recurrent endometrial cancer, and patients with advanced or recurrent endometrial cancer with molecular type dMMR may be much more sensitive to drugs than patients with molecular type pMMR. In our analysis results, patients with advanced or recurrent endometrial cancer classified as dMMR had a lower probability of adverse events than patients with pMMR. In future studies, we should include more study subjects and balance the number of pMMR and dMMR, and inclusion of patients with dMMR and combination studies may be useful.

## Data Availability

The original contributions presented in the study are included in the article/Supplementary Material, further inquiries can be directed to the corresponding author.
